# Characterization of Fungal Species Isolated from Cankered Apple Barks Demonstrates the *Alternaria alternata* Causing Apple Canker Disease

**DOI:** 10.3390/jof10080536

**Published:** 2024-07-31

**Authors:** Zhiqiang Li, Hao Li, Jiating Zhang, Shikai Zhang, Qi Zhao, Chunzhen Cheng, Yongyan Zhang

**Affiliations:** College of Horticulture, Shanxi Agricultural University, Jinzhong 030801, China

**Keywords:** apple (*Malus domestica*), *Alternaria alternata*, *Cytospora*, pathogen isolation, molecular identification

## Abstract

Apple canker disease, also named as apple *Valsa* canker, is one of the most destructive diseases for apples (*Malus domestica* Borkh.). *Cytospora*/*Valsa* spp. are the dominant causal agent of this disease, but many studies have revealed that fungi from some other genus can also cause typical apple canker symptoms. In this study, we performed fungal pathogen isolation from cankered ‘Fuji’ apple barks. Six representative morphologically different fungi (Strain 1–6) were further subjected to *ITS* sequencing and evolutionary analysis. Molecular identification results revealed that Strains 1–6 are *Cytospora mali*, *Fusarium* cf. *solani*, *Alternaria alternata*, *C. mali*, *Diplodia seriata* and *F. proliferatum*, respectively. All these fungi have been reported to be causal agents of apple diseases. By inoculating fungal plugs onto trunks of ‘Fuji’ apple trees, the pathogenicity of the six fungi were accessed. Only the inoculations of the two *C. mali* strains (Strain 1 and Strain 4) and the *A. alternata* strain (Strain 3) resulted in typical apple canker symptoms in trunks. It is worth noting that Strain 1 caused much more severe canker symptoms and higher pathogenicity incidence than the other two fungi. *A. alternata* has been identified as a pathogen causing diseases on apple fruits and leaves. By further assessing its pathogenicity on apple fruits and leaves, we verified that it can also cause typical fruit rot and leaf spot symptoms. To the best of our knowledge, this is the first report on apple canker disease caused by *A. alternata* in China. Our present study can provide a theoretical foundation for the prevention and control of apple canker disease.

## 1. Introduction

Apple (*Malus domestica* Borkh.), the most economically and commercially important temperate fruit crop, is one of the most widely cultivated fruit trees worldwide [1,2]. Its annual production ranked as the fourth of all fruit crops behind banana, orange and grape [3]. China has the largest apple planting area in the world, and produces approximately a half of the world’s apple supply [4,5]. However, in recent years, the apple industry in China has been greatly threatened by some progressive and destructive diseases, such as apple canker disease (also called apple *Valsa* canker disease, AVC), apple ring rot, apple mosaic disease, apple rust disease, apple black rot disease and so on [6,7,8]. Among them, AVC is the most destructive fungal disease [7,9], and has significantly decreased yields of apples in China and worldwide [10]. AVC was first reported in Japan in 1903 [11]. In China, this disease was first reported in Liaoning province in 1916 [12]. Since then, it has emerged as one of the largest troubles for the apple industry in China, causing significant economic losses.

**Figure 1:**
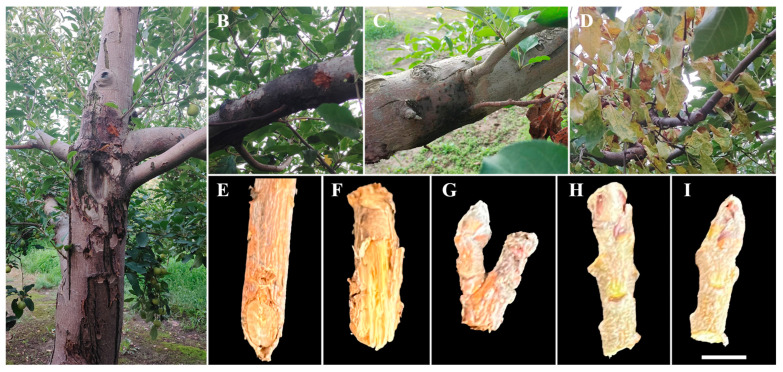
Typical apple canker disease symptoms (**A**–**D**) and cankered apple shoot and bud samples used for pathogen isolation in this study (**E**–**I**). (**A**): typical canker symptom on apple trunk; (**B**,**C**): cankers on branch surface; (**D**): dieback of twigs; (**E**): severe cankered apple shoot; (**F**): severe cankered apple shoot with cracked bark; (**G**): severe diseased bud; (**H**,**I**): mild diseased buds with slight reddish-brown cankers. Scale bar = 0.5 cm (for (**E**–**I**)).

The typical symptoms of AVC mainly include cankers on trunks, branch surfaces and scaffold limbs, dieback of twigs, and death of distal parts and even the whole tree [13,14] (Figure 1). Its effected area develops very fast, especially between spring and early summer [15]. As a disease that has been discovered and reported for more than 120 years, the causal pathogens of apple canker disease have been intensively investigated. Evidence revealed that various *Cytospora*/*Valsa* species are causal agents of AVC [10,14], and *Cytospora*/*Valsa mali* is generally associated with the severest branch and tree deaths [10]. Research also revealed that the *Cytospora* species causing AVC differed in different apple planting areas [16]. In the Tarim Basin of China, *C. pyri* was reported to be the most dominant species causing apple canker disease. *C. melnikii*, *C. tritici*, *C. euonymina* and *C. parasitica* have also been described as causal agents of apple canker in China [17,18]. In apple production hubs of Iran, however, *C. cincta* was identified as an agent causing canker disease. Moreover, fungi from some other genus, such as *Diplodia seriata* [17], *Diplodia bulgarica* [19], *Botryosphaeria dothidea* [20], *Neofusicoccum parvum*, *Diaporthe feoniculina*, *Diaporthe eres*, *Pestalotiposis funerea* and *Phomopsis* spp. [21], have also been reported to have the pathogenicity of causing apple canker. The diversity of causal pathogens makes the control of apple canker disease very hard and to some extent leads to the fact that this disease is still prevalent in many apple producing areas [22].

Shanxi province is one of the seven major apple-planting provinces in China [23]. High incidence of apple canker disease has greatly influenced the apple yield and quality there, especially in Yuncheng city, which is the main apple producing area in Shanxi province. In this study, to investigate the pathogens causing canker disease in ‘Fuji’ apple trees planted in Linyi county of Yuncheng city, fungal isolation and purification, molecular identification and pathogenicity assay experiments were performed. The results obtained in this study will be helpful in guiding pesticide selections for the prevention and control of apple canker disease.

## 2. Materials and Methods

### 2.1. Shoot Sampling and Plant Materials

In October 2023, cankered ‘Fuji’ apple shoots with thicknesses of about 0.5 cm were collected in a 15-year-old apple orchard (35°12′33.02″ N; 110°44′15.01″ E) located in Linyi County, Yuncheng city, Shanxi Province of China. Then, shoots were taken back to lab on ice and used for fungal isolation and identification.

For apple canker disease pathogenicity assays, one-year-old ‘Fuji’ apple seedlings (grafting on *Malus robusta* (CarriŠre) Rehd. rootstocks) were used. To investigate the pathogenicity of *Alternaria alternata* in apple fruits and leaves, ‘Fuji’ fruits and ‘Gala’ leaves were used, respectively. All these apple samples used for pathogenicity assays were provided by our lab.

### 2.2. Isolation and Morphological Identification of Pathogens

Pathogen isolation was performed according to the method of Wang et al. [24]. Bark, at the diseased and healthy conjunction, were cut into about 0.3 cm × 0.3 cm small pieces. After surface sterilizing using 75% ethanol (for 180 s) and 3% sodium hypochlorite solution (for 60 s), samples were washed with sterile water for 30 s 3 times. Then, bark was placed on potato dextrose agar (PDA) medium (containing 30 μg/mL streptomycin) in the dark for two days. Hyphae growing out from the bark were picked and transferred into fresh sterilized PDA media for 2–3 rounds of purifications. Purified fungi were inoculated onto the center of PDA dish (with diameter of 9 cm) to observe their colony characteristics. Colony dimeters of purified fungi were detected every day until colonies covered the whole dish. For each fungus, at least three replications were made. For the morphological observations of mycelia and conidia, fungi that have been cultured on PDA plates for ten days were washed from PDA medium using sterile water, and observed under a microscope (ECLIPSE Ni-U, Nikon, Tokyo, Japan).

### 2.3. Molecular Identification of Isolated Fungi

After culturing on PDA plate at 28 °C for 7 d, mycelia were collected and fast frozen in liquid nitrogen. Then, mycelia were ground into fine powders and subjected to total genomic isolation using the fungus genomic DNA extraction kit (Hangzhou Boer technology Co., Ltd., Hangzhou, China). After DNA quality detection using a NanoONE instrument (Yooning, Hangzhou, China) and 1% agarose gel electrophoresis, DNA samples were diluted into a final concentration of 50 ng/µL and used for *internal transcribed sequence* (*ITS*) fragment amplifications using ITS1/ITS4 primer pair [24,25,26]. The 50 µL PCR system contains 25 µL Dream Taq^TM^ Green PCR Master Mix (2×) DNA polymerase, each of 2 µL ITS1 and ITS4 primer, 19 µL ddH_2_O and a 2 µL DNA template. Amplification was conducted as follows: 95 °C, 3 min; 95 °C, 30 s; 55 °C, 30 s and 72 °C, 30 s, 38 cycles; and 72 °C, 10 min. Amplified PCR products were gel-purified and sent to Sangon Biotech company (Shanghai, China) for Sanger sequencing.

### 2.4. Phylogenetic Analysis

After BLASTn searches in NCBI (https://www.ncbi.nlm.nih.gov/, accessed on 30 October 2023), the top 4~6 homologous *ITS* sequences sharing identity greater than 99% and query cover ratios higher than 97% with *ITSs* of isolated fungi that were downloaded. These downloaded homologous *ITSs* and *ITSs* of isolated fungi were first subjected to multiple sequence alignment analysis using ClustalW 2.1. Then, a phylogenetic tree for these *ITS* sequences was constructed using the Maximum Likelihood (ML) method of MEGA11 under default parameters.

### 2.5. Pathogenicity Identification

In November 2023, to demonstrate the pathogenicity of isolated fungi in causing canker disease, their fungal plugs were inoculated onto trunks of one-year-old ‘Fuji’ apple trees. By using a scalpel, grafting incisions with lengths of about 1.5 cm and widths of about 0.5 cm were cut into trunks at 20–30 cm above ground. One fungal plug (with a dimeter of about 0.5 cm) was inserted into the incision with the mycelia side inwards [20]. Plastic film was used to wrap the incisions tightly. ‘Fuji’ apple trees inoculated with PDA plugs were used as controls. For the inoculations of each fungus and PDA control, 10 replications were made. After inoculation, apple trees were kept in a solar greenhouse. Disease incidence and symptoms were observed every two weeks. For the fungi that can cause canker symptoms in inoculated trunks, fungal re-isolation was performed to fulfill their Koch’s postulates.

### 2.6. Alternaria alternata Inoculation on Apple Fruits and Leaves

*A. alternata* is recognized as a pathogen causing apple postharvest fruit rot and leaf spot diseases. To prove the pathogenicity of the isolated *A. alternata* strain, fruit and leaf inoculation experiments were performed. After puncturing the apple fruits and leaves with needles, fungal plugs with a diameter of 0.5 cm were inoculated onto fruits and leaves. PDA plugs were also inoculated onto fruits and leaves as controls. For both the fungal plug and PDA plus inoculations, ten fruits and ten leaves were used. After *A. alternata* inoculation, fruits and leaves were kept in an air-conditioned room at 25 °C with natural light condition. Fruit symptoms were observed and detected at 1, 5 and 10 days post inoculation (dpi), and leaf symptoms were observed and detected at 1, 2 and 3 dpi, respectively.

### 2.7. Statistical Analysis

Results for the colony dimeters of isolated fungi on PDA media and diseased areas caused by fungal inoculations were displayed as mean ± standard deviation (SD) of at least three replications. Excel 2016 (Microsoft, Redmond, WA, USA) was used to calculate the polynomial regression model for the growth of the six fungi on PDA media. SPSS Statistics version 26.0 (IBM Corporation, Armonk, NY, USA) was applied to analyze the significance of the differences of cankered areas caused by different pathogens or at different timepoints by Duncan’s Multiple Range test at the 5% level. Excel 2016 (Microsoft, Redmond, WA, USA) and Origin 2024 (OriginLab, Northampton, MA, USA) software were used for figure drawing.

## 3. Results

### 3.1. Morphological Analysis of Fungal Colonies

The cankers on diseased ‘Fuji’ apple shoots were yellowish-brown or reddish-brown (Figure 1E–G), and the barks of some severe diseased shoots were cracked and easily fell off (Figure 1F). Buds of severe diseased apple shoots were also reddish-brown (Figure 1H), and small reddish-brown cankers can be observed in the buds of mild diseased apple shoots (Figure 1I).

**Figure 2:**
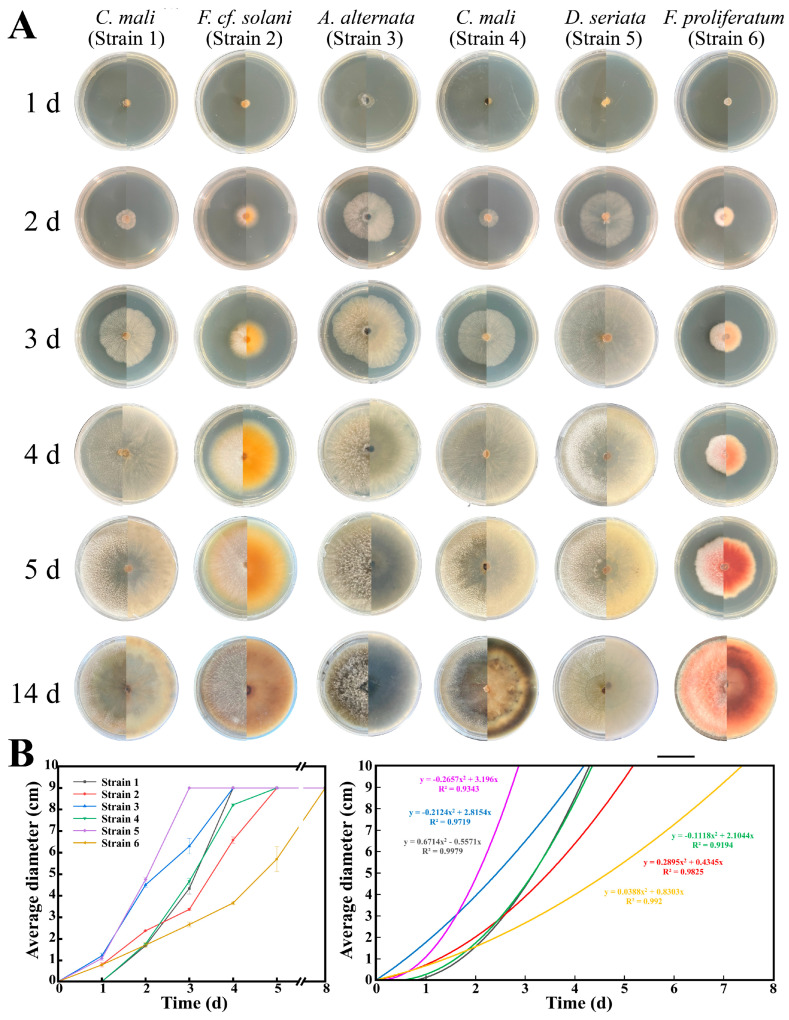
The growth of isolated fungi on PDA media. (**A**): Strain 1–6 cultured on PDA medium for different days; (**B**): growth curve (**left**) and polynomial regression model (**right**) for the growth of the six isolated fungi on PDA media. In (**A**), scale bar = 3 cm.

In total, we isolated and purified 11 fungi from the barks of cankered ‘Fuji’ apple trees. After preliminary morphology observation, six strains (Strain 1–6) were used for further study (Figure 2). The growth rates of these six fungi on PDA media varied a lot (Figure 2A,B). Of them, Strain 5 grows the fastest (able to cover the whole 9 cm PDA dish in approximately three days), followed by Strain 1–4 (in four days), and Strain 6, growing the slowest (in eight days). After 14 days of culture (Figure 2A), the Strain 1 colony is light-yellow and can accumulate yellowish pigments; the Strain 2 colony is orange-yellow and accumulates yellow pigments; the Strain 3 colony is grayish black and accumulates black pigments, and the color of its aerial hyphae gradually change from white to gray or grayish black; the Strain 4 colony is light yellow with grayish-white aerial hyphae and accumulates back pigments; the Strain 5 colony is lilac with grayish-white aerial hyphae and accumulates grayish-white pigments; and the Strain 6 colony is purple-red with white aerial hyphae and accumulates purple-red pigments.

### 3.2. Mycelia and Conidia Observation Results

**Figure 3:**
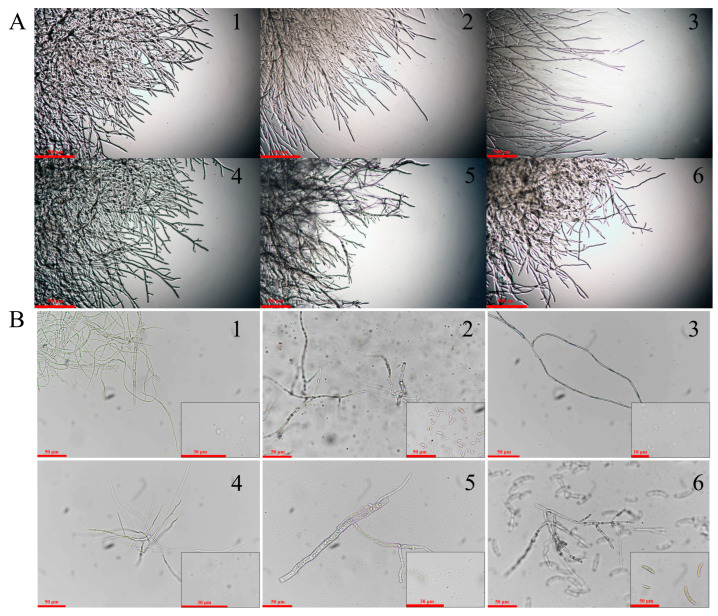
Mycelia and conidia observation results for the six isolated fungi. (**A**): Mycelia at the edge of the PDA dish; (**B**): Morphology of mycelia and conidia. 1–6 is for Strain 1–6, respectively.

**Figure 4:**
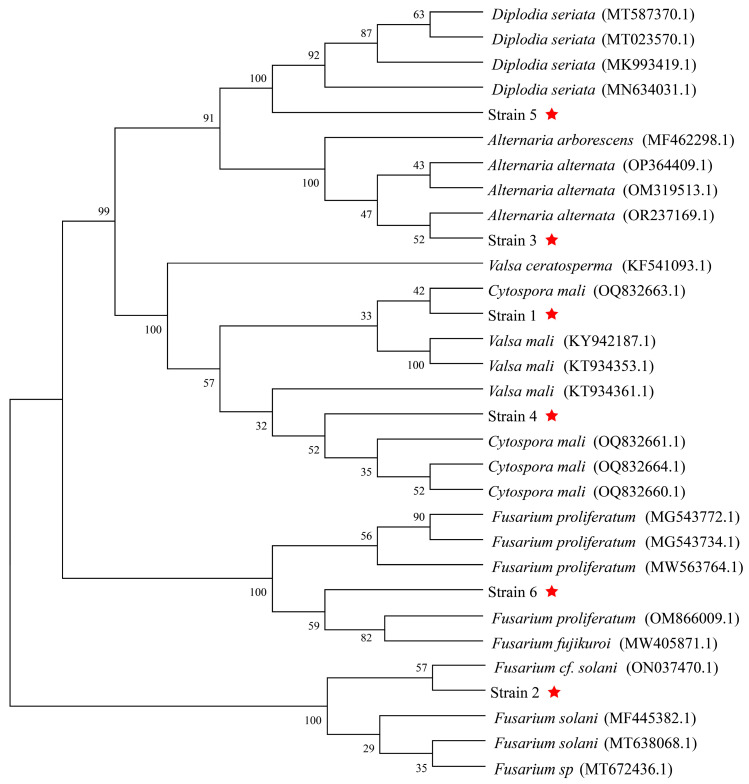
Phylogenetic tree constructed using *ITS* sequences of Strain 1~6 and their homologous sequences. Red five-pointed stars are for the isolated fungi in this study.

**Figure 5:**
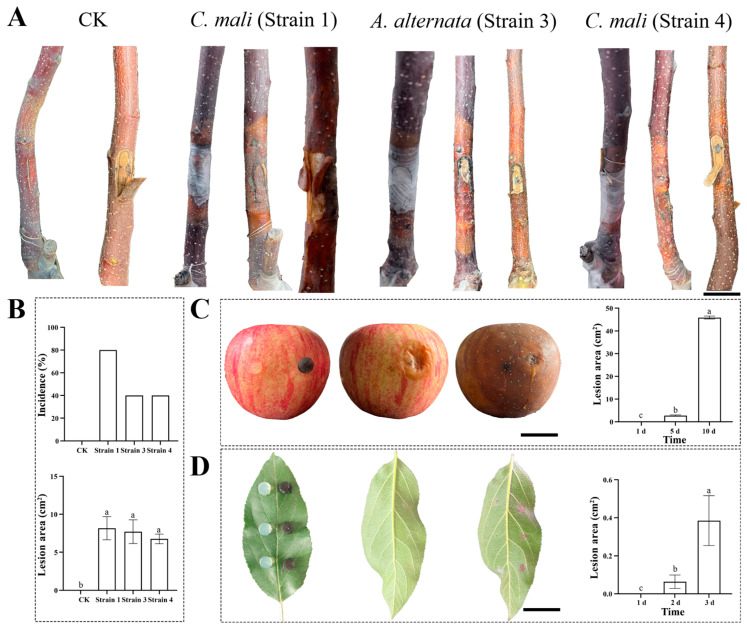
Pathogenicity assay results of Strain 1, Strain 3 and Strain 4. (**A**): canker symptoms caused by Strain 1, Strain 3, and Strain 4. CK is inoculated with PDA plug as control. (**B**): Canker disease incidences and areas caused by Strain 1, Strain 3, and Strain 4. (**C**): Fruit rot symptoms caused by Strain 3. PDA plugs were inoculated on the left part of apples as control. Fruit from left to right is apple at 1, 5 and 10 dpi, respectively. (**D**): Leaf spot symptoms caused by Strain 3. PDA plugs were inoculated on the on the left part of leaves as control. Leaf from left to right was photographed at 1, 2 and 3 dpi, respectively. In (**A**,**C**,**D**), scale bar = 3 cm. In (**B**–**D**), different letters above columns represent significant difference at *p* < 0.05 level.

We further observed the mycelia and conidia of the six fungi. It was found that the mycelia of Strain 1 are septate cylindrical (Figure 3A-1), and its conidia are colorless, round or nearly round (Figure 3B-1). The mycelia of Strain 2 are hollow cylindrical (Figure 3A-2), and its conidia are colorless, nearly oval or sickle shaped (Figure 3B-2). Strain 3 is of septate cylindrical mycelia (Figure 3A-3), and its conidia are single and colorless, round or oval (Figure 3B-3). The mycelium of Strain 4 is hollow cylindrical (Figure 3A-4), and its conidia are single and colorless, round or nearly round (Figure 3B-4). Strain 5 is of septate cylindrical mycelia (Figure 3A-5), and round or oval conidia (Figure 3B-5). Strain 6 is of cylindrical and bamboo-like mycelia (Figure 3A-6) and sickle-shaped or oval, single conidia (Figure 3B-6).

### 3.3. Molecular Identification Results

By using the universal primer ITS1/ITS4, *ITS* sequences of all the six isolated fungi were cloned and sequenced. The *ITS* fragment of Strains 1–6 was 591 bp, 519 bp, 540 bp, 592 bp, 535 bp and 510 bp long, respectively. BLASTn results showed that the *ITS* sequence similarities between Strain 1 and *V. ceratosperma* (KF541093.1), *V. mali* (KT934353.1 and KY942187.1) and *C. mali* (OQ832663.1), between Strain 2 and *Fusarium* cf. *solani* (ON037470.1), *F. solani* (MT638068.1 and MF445382.1) and *F.* sp. (MT672436.1), between Strain 3 and some *Alternaria alternata* (OP364409.1, OM319513.1 and OR237169.1), between Strain 4 and *V. mali* (KT934361.1), *V. ceratosperma* (KF541093.1) and *C. mali* (OQ832660.1, OQ832661.1, OQ832663.1 and OQ832664.1), and between Strain 5 and some *Diplodia seriata* (such as MT587370.1, MK993419.1, MT023570.1 and MN634031.1) are all higher than 99% (Table 1). Moreover, the *ITS* of Strain 6 shares more than 99% similarity with *ITSs* of *F. fujikuroi* (MW405871.1) and several *F. proliferatum* strains (such as MG543772.1, MG543734.1, MW563764.1 and OM866009.1).

A phylogenetic tree was further constructed using these *ITS* sequences (Figure 4). Results revealed that Strains 1–6 are close to *C. mali* (OQ832663.1), *F.* cf. *solani* (ON037470.1), *A. alternata* (OR237169.1), *C. mali* (OQ832661.1), *D. seriata* (MN634031.1), and *F. proliferatum* (OM866009.1), respectively. Therefore, the isolated Strain 1 and Strain 4 are identified as *C. mali*, and the Strains 2, 3, 5 and 6 are identified as *F.* cf. *solani*, *A. alternata*, *D. seriata* and *F. proliferatum*, respectively.

### 3.4. Apple Canker Disease Pathogenicity Assay Results

Fungal plugs of the six candidate fungi were inoculated onto trunks of one-year ‘Fuji’ apple tree to verify their pathogenicity. No obvious disease symptom was found within the early two months after fungal inoculation (from January 2024 to February 2024). However, at three months post inoculation (March 2024), apple trunks inoculated with Strain 1, Strain 3 and Strain 4 showed obvious canker symptoms and these canker areas spread rapidly (Figure 5A), which may be related to the gradual increase of temperature [15]. Typical apple canker disease symptoms, including light brown disease rings at the inoculation site and water-stained infected area, were observed (Figure 5A). However, cracked bark was only found in Strain 1 inoculated apple trunks (Figure 5A). The pathogenicity incidence of Strain 1 was 80% (Figure 5B), accounting for double that of Strain 3 and Strain 4. Moreover, although no significant difference was found among the canker areas caused by the three fungi, the average canker area caused by them followed the order of Strain 1 > Strain 3 > Strain 4 (Figure 5B). These indicated that the pathogenicity of the three causal agents varied. These three fungi were successfully re-isolated and purified from diseased regions, which fulfilled their Koch’s postulates of causing apple canker disease.

### 3.5. Pathogenicity Assay Results of A. alternata in Causing Apple Fruit and Leaf Diseases

To investigate the pathogenicity of *A. alternata* (Strain 3) in causing fruit and leaf diseases, we further conducted inoculation experiments on apple fruits and leaves. Results showed that Strain 3 inoculation led to severe fruit rot (Figure 5C) and leaf spot diseases (Figure 5D). Notably, its causal water-stained rot symptoms spread very fast, being able to rot the whole fruit at 10 dpi (Figure 5C). These results indicated that *A. alternata* can not only cause diseases in apple fruits and leaves, but also canker disease in apple trunks.

## 4. Discussion

In this study, to identify pathogens causing apple canker disease in the Shanxi province of China, fungal isolation and purification, and pathogenicity assays, were performed. The six candidate pathogens identified in our present study all have been reported to be pathogens causing apple diseases. Among them, *C. mali* has been recognized as the dominant pathogen of AVC [18]; *F*. cf. *solani* was reported to be a causal agent of apple replant disease (ARD) [27]; *F. proliferatum* was identified as a pathogen that causes ARD, apple bud-base rot and twig blight [28,29]; *A. alternata* has been reported to be a causal pathogen for apple *Alternaria* blotch disease, calyx rot, core rot, and so on [30,31,32]; and *D. seriata* has been reported to cause apple cankers and dieback, postharvest apple fruit decay, black rot canker and black spot diseases [33,34,35,36,37]. However, only the Koch’s postulates of *C. mali* (Strain 1 and Strain 4) and *A. alternata* causing apple canker disease were completed in our study, indicating that these three fungi are the causal agents of the apple canker disease there.

*Cytospora* can cause canker or dieback diseases in more than 130 woody plant species [38]. Among them, apple canker disease caused by *Cytospora/Valsa* species is the severest disease threatening the apple industry worldwide [16]. Although the *Cytospora/Valsa* species causing canker disease in different apple producing areas varied, *Cytospora/Valsa mali* was usually identified as the most dominant [39]. In this study, we confirmed that two *C. mali* strains (Strain 1 and Strain 4) are causal agents of apple canker disease in Shanxi province of China. Moreover, we found that the pathogenicity incidence of Strain 1 is higher than that of Strain 4, and the cankered symptoms caused by it are also more severe than Strain 4. This indicated that the pathogenicity incidences and symptoms caused by different *C. mali* strains varied. In addition to *C. mali*, some other *Cytospora* spp. have also been reported to have the ability to cause apple canker disease. For example, Azizi et al. [40] found that *C. balanejica* could infect several apple varieties and cause sunken and discolored bark and wood. They also found that the pathogenicity of *C. balanejica* varied in different apple varieties, i.e., causing the severest disease symptoms in ‘M4’ apple plants and the mildest symptoms in ‘Golden Delicious’ apple plants. Hanifeh et al. [41] reported that *C. avicennae*, *C. azerbaijanica*, *C. ershadii*, *C. iranica*, *C. chrysosperma*, *C. parasitica* and *C. Parasitica*, can all cause AVC on apple plants.

In addition to *Cytospora*/*Valsa*, *Diplodia* species have also been identified as pathogens causing apple canker disease [30]. *D. intermedia* was the main pathogen causing AVC in commercial orchards in southwestern Ontario of Canada [42]. *D. seriata* infection would lead to smoky canker symptoms in Indian apple trees [34], such as water soaked incisions, and yellow to brown discoloration of incision sites and so on. However, in our study, no apple canker disease symptom was caused by our isolated *D. seriata* strain in inoculated trunks of ‘Fuji’ apple plants, indicating that *D. seriata* is not a causal agent of apple canker disease in this apple variety. There are also some reports about the apple canker disease causal fungi from some other genus. For example, Karlstrom et al. [43] found that *Neonectria ditissima* can cause canker in apples and other broad-leaved trees.

In China, the main pathogens causing apple canker disease were reported to be *Cytospora*/*Valsa mali* [1,44]. Liang et al. [45] reported that *C. mali* weakens apple trees and significantly reduces apple production in China and other East Asian countries. Zhao et al. [46] confirmed that *C. mali* could infect ‘Jin hong’ apples, and their infected tissues would gradually dry out, collapse slightly, and finally form localized cankers and even whole apple tree death. In recent years, canker disease has seriously influenced the yield and quality of apples in Shanxi province of China. In Mengxian County of Shanxi Province, *V. mali* is identified as a pathogen causing apple canker disease [47]. In this study, we found that the causal pathogens in cankered ‘Fuji’ apples in Linyi County, Yuncheng city of Shanxi Province included two *C. mali* strains and one *A. alternata* strain. This suggests that the prevention and control methods for apple canker disease in different apple producing areas should not be the same.

*A. alternata* can cause a variety of plant diseases [48]. At present, it has been reported to be pathogens causing wolfberry root rot disease [49], kiwifruit soft rot [50], pitaya fungal stem end rot disease [51], cotton stem blight [52], jujube rot disease [53], cherry tomato black rot disease [54], and so on. In apples, *A. alternata* has also been proven to be associated with several fungal diseases, including apple *Alternaria* blotch disease [55], postharvest fruit rot [56], fruit body rot and calyx rot [57]. Our study revealed that *A. alternata* can not only cause apple fruit rot and leaf spot diseases, but can also cause apple canker disease. To the best of our knowledge, this is the first report on apple canker disease caused by *A. alternata* in China. Therefore, attention should also be paid to *A. alternata* in preventing and controlling apple canker disease in the future.

## 5. Conclusions

In this study, to provide a basis for the prevention and control of apple canker disease in Shanxi province, we performed causal fungal pathogen isolation and identification experiments. Two *C. mali* strains were identified as causal agents of apple canker disease. Moreover, for the first time, *A. alternata* was demonstrated to be a causal agent of apple canker disease in China. Our study can provide a basis for the prevention and control of apple canker disease and can be helpful in guiding pesticide selection and application for controlling this disease.

## Figures and Tables

**Table 1:** Blastn results for the *ITSs* of the six isolated fungi.

Strain	Scientific Name	Query Cover Ratio	E-Value	Identity	Genbank Accession No.
Strain 1	*Cytospora ceratosperma*	97.00%	0	100.00%	KF541093.1
	*Cytospora mali*	99.00%	0	99.49%	OQ832663.1
	*Valsa mali*	99.00%	0	99.49%	KY942187.1
	*Valsa mali*	99.00%	0	99.49%	KT934353.1
Strain 2	*Fusarium solani*	98.00%	0	100.00%	MT638068.1
	*Fusarium solani*	98.00%	0	100.00%	MF445382.1
	*Fusarium* cf. *solani*	98.00%	0	100.00%	ON037470.1
	*Fusarium sp.*	98.00%	0	100.00%	MT672436.1
Strain 3	*Alternaria alternata*	97.00%	0	100.00%	OP364409.1
	*Alternaria alternata*	97.00%	0	100.00%	OM237169.1
	*Alternaria arborescens*	98.00%	0	99.81%	MF462298.1
	*Alternaria alternata*	100.00%	0	99.45%	OM319513.1
Strain 4	*Valsa mali*	99.00%	0	100.00%	KT934361.1
	*Cytospora ceratosperma*	97.00%	0	100.00%	KF541093.1
	*Cytospora mali*	99.00%	0	100.00%	OQ832663.1
	*Cytospora mali*	99.00%	0	99.66%	OQ832664.1
	*Cytospora mali*	99.00%	0	99.66%	OQ832660.1
	*Cytospora mali*	99.00%	0	99.49%	OQ832661.1
Strain 5	*Diplodia seriata*	99.00%	0	99.44%	MT587370.1
	*Diplodia seriata*	99.00%	0	99.44%	MN634031.1
	*Diplodia seriata*	99.00%	0	99.44%	MT023570.1
	*Diplodia seriata*	99.00%	0	99.44%	MK993419.1
Strain 6	*Fusarium proliferatum*	97.00%	0	100.00%	MG543772.1
	*Fusarium proliferatum*	97.00%	0	100.00%	MG543734.1
	*Fusarium proliferatum*	97.00%	0	100.00%	MW563764.1
	*Fusarium proliferatum*	97.00%	0	100.00%	OM866009.1
	*Fusarium fujikuroi*	97.00%	0	100.00%	MW405871.1

## Data Availability

The raw data supporting the conclusions of this article will be made available by the authors on request.
